# Dignity of Risk Attuned Care: A Knowledge Translation Initiative via Education With Healthcare Staff

**DOI:** 10.1093/geroni/igaf122.2088

**Published:** 2025-12-31

**Authors:** Catherine-Anne Murray, Kailey Durette

## Abstract

The Dignity of Risk approach is an important human rights-based approach to older adult care. It recognizes that daily life is inherently uncertain and taking risks can lead to positive outcomes in quality of life, health and well-being. When living with frailty, dementia or changing abilities, older adults can be supported to take reasonable risks that uphold their preferences and values. Unfortunately, current healthcare practice with older adults poses challenges to the implementation of Dignity of Risk principles. These can include ageism, overprotective attitudes, misconceptions regarding liability, and a hyperfocus on “keeping safe”. These underlying biases and attitudes contribute to an interdisciplinary care culture that can unintentionally restrict and remove autonomy and independence of older adults. This practice-based research and education initiative aims to: 1. Explore healthcare staff’s attitudes, beliefs, barriers, and facilitators surrounding the application of Dignity of Risk to care 2. Develop and deliver education workshops that aim to enhance staff’s knowledge, awareness, and skills in integrating Dignity of Risk principles into care of older adults. 3. Use key findings and lessons learned to inform evolution of ongoing education methods and implications to healthcare policy and quality improvement. Understanding how to shift culture towards supporting older adults and their caregivers using a risk management approach to care planning can have a positive effect not only on maintaining older adults’ quality of life, wishes, and values, but also on the healthcare system as it faces increasing demands to meet the needs of older adults living with frailty.

